# Associations of eccentric force variables during jumping and eccentric lower-limb strength with vertical jump performance: A systematic review

**DOI:** 10.1371/journal.pone.0289631

**Published:** 2023-08-03

**Authors:** Daichi Nishiumi, Takuya Nishioka, Hiromi Saito, Takanori Kurokawa, Norikazu Hirose

**Affiliations:** 1 Graduate School of Sport Sciences, Waseda University, Saitama, Japan; 2 Institute of Physical Education, Keio University, Hiyoshi, Yokohama, Japan; 3 Faculty of Sport Sciences, Waseda University, Saitama, Japan; Federation University Australia, AUSTRALIA

## Abstract

The purpose of this systematic review was to summarize the associations of eccentric force variables during jumping and eccentric lower-limb strength with vertical jump performance. A literature search was conducted in September 2022 using PubMed, Web of Science, and Scopus. Thirteen cross-sectional studies investigating the relationship between eccentric force and strength variables, such as force, rate of force development (RFD), power, time, and velocity, and vertical jump performance, including the jump height, reactive strength index (RSI), and reactive strength index-modified (RSImod), were included in this systematic review. As eccentric strength, variables during the unloading-to-braking phase of countermovement jump (CMJ) (force, RFD, etc.) and the eccentric force of the squat movement and knee joint were included. The CMJ height, RSImod, and drop jump RSI were included to analyze the vertical jump performance. The modified form of the Downs and Black checklist was used to evaluate quality. Associations between the force and RFD during the descending phase of the CMJ and jump height were observed in some studies but not in others, with differences between the studies. Some studies reported associations between the force and/or RFD during the descending phase of the CMJ and RSImod of the CMJ, with no differences among their results. In addition, there are associations of the eccentric forces during squatting and knee extension with the CMJ and the drop jump heights and RSI of the drop jump. The eccentric force variables in the CMJ and RSImod are related; however, their relationship with jump height remains unclear. Furthermore, improved eccentric muscle strength may contribute to vertical jump height because of the associations of the eccentric strength during knee extension and squatting with jump height.

## Introduction

Improving vertical jump performance is becoming a topic of traditional interest in competitive sports. Jump height is associated with sprinting [1] and change of direction (COD) ability [2], as well as competitive-level in volleyball [3], soccer [4], and basketball [5]. The reactive strength index (RSI = jump height/contact time) has also been studied as an indicator of vertical jump performance and is associated with COD ability [6]. Furthermore, the reactive strength index-modified (RSImod = jump height/jump time) has been recently studied and is considered an important ability, especially for volleyball and basketball [7, 8].

In a vertical jump with a stretch-shortening cycle [e.g., countermovement jump (CMJ) and drop jump], jump height is determined by the concentric phase impulse according to Newton’s second law of motion. In this regard, the key focus for improving CMJ is increasing the concentric phase impulse. The impulse can be improved by applying a large force during the early concentric phase and maintaining it until the late concentric phase. Therefore, training to increase the initial (i.e., rate of force development: RFD) and peak concentric forces is being actively undertaken [9].

The use of a countermovement prior to the concentric movement generates a large force during the downward-transition phase, which is another method to improve the early concentric phase force [10]. This may improve the total concentric phase impulse, which would in turn improve jump height. Furthermore, a greater force applied during the downward phase shortens the downward and amortization phase durations, which may also contribute to the RSI and RSImod. Since the downward phase involves a stretched muscle exerting force, the mechanical variables during the countermovement (i.e., downward phase) may be influenced by the lower extremity eccentric muscle strength, which may simultaneously contribute to the jump performance [11].

The importance of exerting force during the concentric phase has been established by Newton’s second law and previous review studies [12]. However, the relationship between the eccentric variables, such as downward phase force and eccentric muscle strength, and vertical jump performance has not been previously summarized. Therefore, the purpose of this study was to summarize the associations of eccentric force variables during jumping and eccentric lower-limb strength with vertical jump performance. This review will help practitioners understand the importance of improving the eccentric variables as well as the concentric ones to improve their athletes’ vertical jump performance.

## Materials and methods

### Search strategy and information sources

This systematic review followed the preferred reporting items for systematic reviews and meta-analyses 2020 guidelines [13]. As this study did not involve human participants, institutional review board approval was not required. A literature search was conducted by one author on September 30th, 2022 using the PubMed, Web of Science, and Scopus databases. The search terms are listed in Table 1.

**Table 1:** Terms used during the literature search.

Terms
(“eccentric strength” or “eccentric force” or “eccentric power” or “eccentric velocity” or “eccentric impulse” or “eccentric moment” or “eccentric torque” or “eccentric time” or “eccentric duration” or “braking strength” or “braking force” or “braking power” or “braking velocity” or “braking impulse” or “braking moment” or “braking torque” or “braking time” or “braking duration” or “negative strength” or “negative force” or “negative power” or “negative velocity” or “negative impulse” or “negative moment” or “negative torque” or “negative time” or “negative duration” or “downward strength” or “downward force” or “downward power” or “downward velocity” or “downward impulse” or “downward moment” or “downward torque” or “downward time” or “downward duration” or “descending strength” or “descending force” or “descending power” or “descending velocity” or “descending impulse” or “descending moment” or “descending torque” or “descending time” or “descending duration” or “deceleration strength” or “deceleration force” or “deceleration power” or “deceleration velocity” or “deceleration impulse” or “deceleration moment” or “deceleration torque” or “deceleration time” or “deceleration duration”) and ("jump")

### Eligibility criteria

The PECO (Population, Exposure, Comparator, and Outcome) questions for this study were as follows: Population, athletes aged 16–40 years; Exposure, eccentric force and strength; Comparator, jump performance; and Outcome, correlation coefficient (r) or effect size (Cohen’s d). The inclusion criteria for this study were as follows: 1) written in English, 2) cross-sectional study design, 3) healthy athletes aged 16–40 years, 4) measurement of eccentric muscle strength and/or force and/or time and/or velocity and/or power and/or RFD and/or impulse, and 5) measurement of jump height and/or RSI and/or RSImod for countermovement, drop, or depth jump.

The exclusion criteria for this study were as follows: 1) intervention studies, 2) competition-specific jumping movements (for example, high jump), 3) no correlation analysis or group comparisons conducted, and 4) in group comparison studies, no grouping conducted on the basis of eccentric force and strength or jump performance.

### Study selection

The titles and abstracts of the literature works obtained from the databases were independently screened by three researchers. Subsequently, the full text of the selected literature was independently screened by the three researchers. Any disagreements among the three were resolved by consensus.

### Quality assessment (Risk of bias)

The quality assessment of the research articles was conducted independently by two researchers using the modified form of the Downs and Black checklist [14]. This checklist has been previously used with adjustments for cross-sectional studies; it was similarly used in this review [6, 15]. The checklist has 13 items (1, 2, 3, 5, 6, 7, 10, 11, 12, 16, 18, 20, and 25), with a maximum score of 14 points. Only number 5 on the checklist has a maximum score of 2 (2 = “yes,” 1 = “partially,” and 0 = “no”). Scores of ≥ 10, 5–9, and ≤ 4 indicated high, moderate, and low quality, respectively. After the independent quality assessments, a consensus meeting was held to determine the final scores.

### Data collection and data items

Table 2 summarizes the main results of the included studies. The topics in Table 2 are defined as follows: (1) study, (2) group, (3) number of subjects, (4) age, (5) type of sports, (6) eccentric variables, (7) type of jump, (8) jump performance, (9) measurement method, (10) countermovement strategies and (11) main results.

**Table 2:** Summary of the main results of the studies included in this review.

Study	Group	Number of subjects	Age (mean ± SD)	Type of sports	Type of exercise	Eccentric variables	Type of jump	Jump performance	Measurement method	Countermovement strategy	Arm swing strategy	Main results	
González -Badillo and Marques [29]		48	22.5	Trained, Track and field (sprinters, long and triple jumpers, indoor hard court)	CMJ	Unloading ~ yielding force	loaded CMJ (17kg)	Jump height	N/A	N/A	Grip the barbell	*r* = 0.67–0.584	*p* < 0.05
						Unloading ~ yielding time						*r* = −0.335	*p* < 0.05
												*r* = −0.297	*p* < 0.05
												*r* = −0.328	*p* > 0.05
						Unloading ~ yielding impulse						*r* = 0.28	*p* > 0.05
												*r* = 0.412	*p* < 0.05
												*r* = 0.384	*p* < 0.05
						Unloading ~ yielding peak power						*r* = 0.545–0.675	*p* < 0.001
						Unloading ~ yielding average power						*r* = 0.582–0.684	*p* < 0.001
						Braking force						*r* = 0.741–0792	*p* < 0.001
						Braking impulse						*r* = 0.367–0.397	*p* < 0.01
						Braking peak power						*r* = 0.423–0.66	*p* < 0.01
						Braking average power						*r* = 0.609–0.754	*p* < 0.001
						Maximum negative velocity						*r* = 0.542–0.649	*p* < 0.001
Laffaye et al. [19]		273	28	Trained, Basketball, Football, Volleyball, Baseball, Others	CMJ	Braking RFD	CMJ	Jump height	Impulse-momentum method	Uncontrolled	With arm swing	*r* = 0.52	*p* < 0.01
						Relative braking RFD		Jump height	Impulse-momentum method			*r* = 0.40	*p* < 0.01
						Eccentric time		Jump height	Impulse-momentum method			*r* = −0.21	*p <* 0.01
						Ratio of eccentric time to jump time		Jump height	Impulse-momentum method			*r* = −0.23	*p <* 0.01
Floría et al. [20]	High jump group	17	17.8 ± 1.2	Rugby	CMJ	Braking force	CMJ	Jump height	Impulse-momentum method	Uncontrolled	Hands on hips	*d* ≥ 0.7	*p* ≤ 0.049
	Low jump group	17	17.5 ± 0.9			Late of braking RFD						*d* ≥ 0.7	*p* ≤ 0.046
						Downward velocity						*d* ≥ −0.7	*p* ≤ 0.049
Bridgeman et al. [28]		12	25.4 ± 3.5	Strength-trained athletes	Squat	Eccentric force	CMJ	Jump height	N/A	Uncontrolled	With arm swing	*r* = 0.73	*p* ≤ 0.001
González-Moro et al. [11]		13	34.4 ± 4.4 (male)	Acrobatic skydivers	Q 60°/s ecc H 60°/s ecc Q 180°/s ecc H 180°/s ecc	Isokinetic eccentric force	CMJ	Jump height	Flight-time method	90° knee-flex	Hands on hips	*r* = 0.658 (Q 60°/s ecc)	*p* = 0.011
												*r* = 0.518 (H 60°/s ecc)	*p* = 0.07
			35 ± 2.6 (Female)									*r* = 0.737 (Q 180°/s ecc)	*p* = 0.004
												*r* = 0.436 (H 180°/s ecc)	*p* = 0.137
Harry et al. [26]	Good RSImod	6	24 ± 1	Recreationally active	CMJ	Eccentric RFD	CMJ	RSImod	Jump height divided by jump time	Uncontrolled	With arm swing	*d* = 0.03	*p* = 0.953
	Poor RSImod	9	24 ± 3			Unloading time						*d* = 1.08	*p* = 0.04
						Eccentric time						*d* = 0.55	*p* = 0.427
Barker et al. [21]		26	19.65 ± 1.23	Soccer	CMJ	Unloading force	CMJ	Jump height	Impulse-momentum method	Uncontrolled	Hands on hips	*r* = -0.101	*p* > 0.05
								RSImod	Jump height divided by jump time			*r* = -0.467	*p* < 0.05
						Unloading RFD		Jump height	Impulse-momentum method			*r* = 0.018	*p* > 0.05
								RSImod	Jump height divided by jump time			*r* = -0.246	*p* > 0.05
						Eccentric work		Jump height	Impulse-momentum method			*r* = 0.125	*p* > 0.05
								RSImod	Jump height divided by jump time			*r* = 0.607	*p* < 0.05
						Eccentric RFD		Jump height	Impulse-momentum method			*r* = 0.097	*p* > 0.05
								RSImod	Jump height divided by jump time			*r* = 0.755	*p* < 0.05
						Amortization force		Jump height	Impulse-momentum method			*r* = 0.11	*p* > 0.05
								RSImod	Jump height divided by jump time			*r* = 0.725	*p* < 0.05
Douglas et al. [22]		13 (team sport athletes)	23 ± 3	Trained, Hockey, Rugby, Soccer, Track-and-field sprinters	Back squat	Isoinertial eccentric force	DJ from 50 cm	RSI	Flight time divided by contact time	N/A	Hands on hips	*r* = 0.6 (90% CI: 0.31–0.79)	*p* < 0.05
		11 (track-and-field sprinters)	23 ± 5										
Merino-Muñoz et al. [23]		21	24.4 ± 4.0	Soccer	CMJ	Braking RFD	CMJ	Jump height	Flight-time method	Uncontrolled	Hands on hips	*r* = 0.319	*p* = 0.158
Krzyszkowski et al. [8]	High RSImod	11	20.5 ± 2	Basketball	CMJ	Unloading RFD	CMJ	RSImod	Jump height divided by jump time	N/A	With arm swing	*d* = 0.22	*p* = 0.607
	Low RSImod	11	20 ± 2			Braking RFD						*d* = 1.41	*p* = 0.005
McHugh et al. [24]	Below average jump height	19	21 ± 3	hockey, lacrosse, soccer, basketball, track, field hockey, football, rugby, and skiing	CMJ	Unloading ~ yielding time	CMJ	Jump height	Impulse-momentum method	Uncontrolled	Hands on hips	*d* = 0.13	*p* > 0.05
						Minimum force						*d* = 0.64	*p* > 0.05
						Braking peak power						*d* = 1.25	*p* < 0.05
						Amortization force						*d* = 1.20	*p* < 0.05
	Above average jump height	17				Braking RFD						*d* = 1.15	*p* < 0.05
						Braking time						*d* = 0.99	*p* < 0.05
						Eccentric force						*d* = 1.19	*p* < 0.05
Harper et al. [25]		29	19.7 ± 1.8	Soccer, Rugby	DJ 20 cm	Eccentric mean force	DJ 20 cm	RSI	Jump height divided by contact time	N/A	Hands on hips	*r* = 0.40 (90% CI: 0.10–0.63)	*p* > 0.05
								Jump height	Flight-time method			*r* = −0.05 (90% CI: −0.36–0.27)	*p* > 0.05
							DJ 40 cm	RSI	Jump height divided by contact time			*r* = 0.11 (90% CI: −0.21–0.41)	*p* > 0.05
								Jump height	Flight-time method			*r* = −0.17 (90% CI: −0.45–0.15)	*p* > 0.05
					DJ 40 cm		DJ 20 cm	RSI	Jump height divided by contact time			*r* = 0.32 (90% CI: 0.01–0.57)	*p* > 0.05
								Jump height	Flight-time method			*r* = −0.04 (90% CI: −0.35–0.28)	*p* > 0.05
							DJ 40 cm	RSI	Jump height divided by contact time			*r* = 0.46 (90% CI: 0.17–0.68)	*p* > 0.05
								Jump height	Flight-time method			*r* = −0.18 (90% CI: −0.47–0.14)	*p* > 0.05
Oh and Lee [27]	High eccentric strength group	6	25.8 ± 2.32	physically active	Knee extension	Eccentric torque	DJ 50cm	Jump height	Impulse-momentum method	N/A	Hands on hips	*d* = 1.02	*p* = 0.047
	Low eccentric strength group	10	25.7 ± 2.58										

CMJ, countermovement jump; Q, quadriceps; H, hamstrings; RFD, rate of force development; DJ, drop jump; RSI, reactive strength index; RSImod, reactive strength index-modified; r, Pearson’s correlation coefficient; d, Cohen’s effect size

### Term definitions for the phases of countermovement jump

Similar definitions for the CMJ phases have been used in the reviewed articles; however, the terms used for each phase were different. Therefore, the terms used in this paper were standardized as follows [16]. The unloading, yielding, braking, eccentric, and concentric phases were defined as the periods from the initiation of the movement to the minimum ground reaction force, minimum ground reaction force to the maximum negative center-of-mass velocity, maximum negative center-of-mass velocity to the center-of-mass velocity reaching 0 m/s, initiation of the yielding phase to the end of the braking phase, and end of the eccentric phase to takeoff, respectively (Table 3).

**Table 3:** Term definitions for the phases of countermovement jump.

Phase	Term definition [16]
Unloading	Initiation of the movement to the minimum ground reaction force
Yielding	Minimum ground reaction force to the maximum negative center-of-mass velocity
Braking	Maximum negative center-of-mass velocity to the center-of-mass velocity reaching 0 m/s
Eccentric	Initiation of the yielding phase to the end of the braking phase
Concentric	End of the eccentric phase to takeoff

### Term definitions for the vertical jump performance

In this study, vertical jump performance was defined as the jump height, RSImod, and RSI of the CMJ and DJ, including the downward phase. The jump height calculation method varied among the studies; the impulse and flight-time method were used in each study. The impulse-momentum and flight-time method calculations were as follows: Vto^2^/2g and gt^2^/8, respectively, where Vto is the takeoff velocity, g is the acceleration due to gravity, and t is the flight time. RSImod and RSI calculations were as follows: jump height/jump time and flight time or jump height/contact time, respectively.

### Interpretation of the effect size

The reported effect sizes for Cohen’s *d* and Pearson’s *r* were interpreted, respectively, as: small: > 0.2, medium: > 0.5, and large: > 0.8 [17]; trivial: < 0.1, small: > 0.1, moderate: > 0.3, high: > 0.5, very high > 0.7, and practically perfect: > 0.9 [18].

## Results

### Study selection

**Fig 1:**
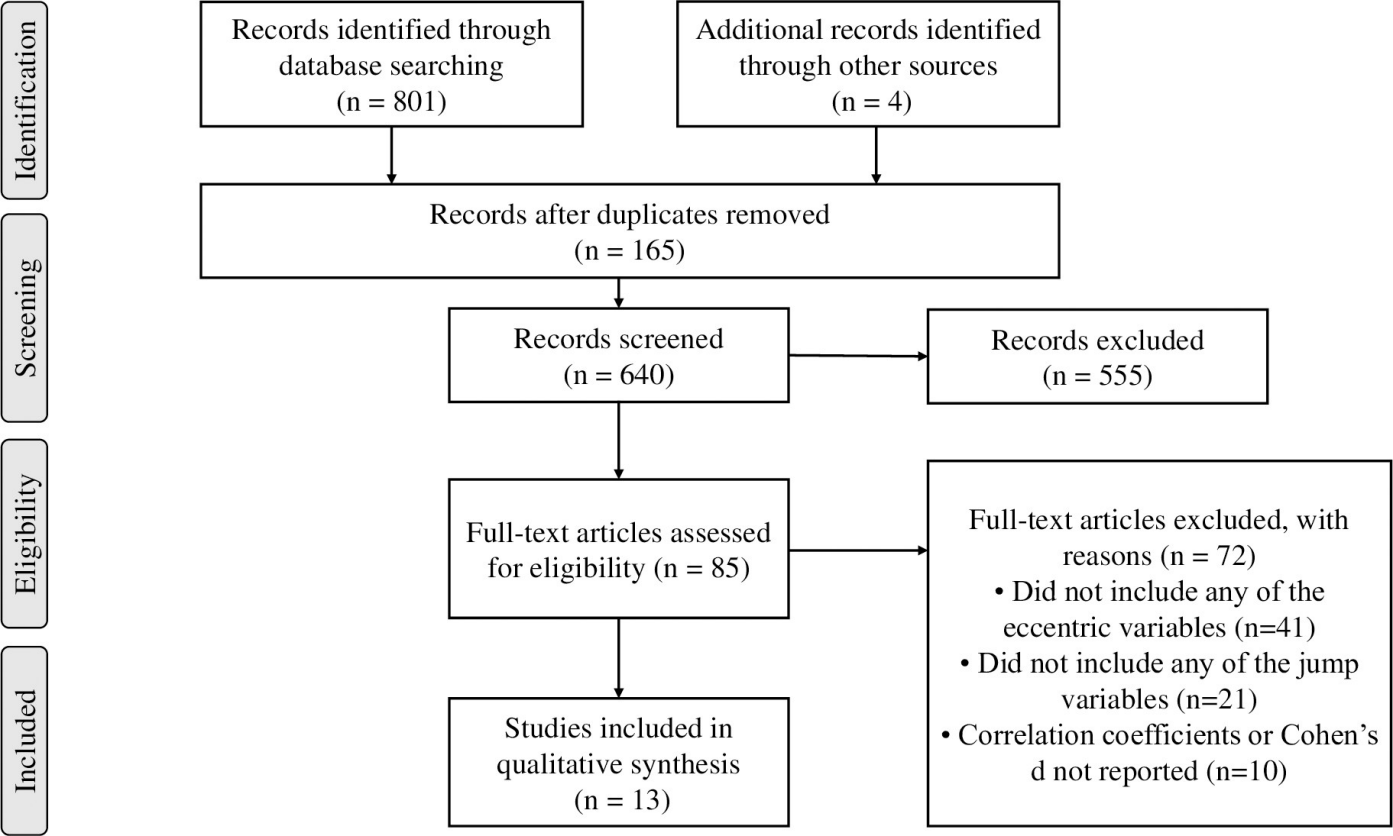
Preferred reporting items for systematic reviews and meta-analyses (PRISMA) flowchart of the study selection process.

We selected 805 studies—801 through database searches (308, 218, and 275 from PubMed, Web of Science, and Scopus, respectively) and four through manual searching. One hundred sixty-five duplicate references were removed, resulting in 640 studies; the title and abstract screening yielded 85 results. Full-text screening yielded 13 studies, which were reviewed in the present study (Fig 1).

### Study characteristics

This review included 13 cross-sectional studies that investigated the relationship between eccentric strength variables and vertical jump performance. Regarding the participant characteristics, eight studies involved team sports athletes [8, 19–25], three involved track-and-field athletes [9, 22, 24], two recreational activity-level participants [26, 27] and others involved skydivers [11], and strength-trained athletes [28]. The team sports included soccer, rugby, basketball, football, volleyball, baseball, and field hockey.

Eccentric strength variables were measured during CMJs in eight studies [8, 19–21, 23, 24, 26, 29], multi-joint movements in two [22, 28], single-joint movements in two [11, 27], and drop jumps in one [25].

As for the jump types, ten studies used CMJs [8, 11, 19–21, 23, 24, 26, 28, 29], and the other three used drop jumps [22, 25, 27].

In the context of jump performance indexes, seven studies measured the CMJ heights [11, 19–21, 23, 24, 28, 29], three measured the RSImod of the CMJ [8, 21, 26], and the other two measured the drop jump RSI and height [22, 25, 27].

Regarding the statistical analysis methods, eight studies investigated relationships and reported the correlation coefficients [11, 19, 21–23, 25, 28, 29], and five studies reported effect sizes with between-group comparisons [8, 20, 24, 26, 27].

### Quality assessment (Risk of bias)

The modified Downs and Black checklist scores ranged from 7 to 12 points: nine studies were considered high quality [8, 19, 21–24, 26, 29, 27], and four were of moderate quality [11, 20, 25, 28]. Table 4 shows the scores for each item in the reviewed studies.

**Table 4:** Risk of bias and quality of the selected studies, with scores for each item on the Downs and Black checklist.

Study	1	2	3	5	6	7	10	11	12	16	18	20	25	Scores	Quality
González-Badillo and Marques	1	1	1	2	1	1	0	0	0	1	1	1	0	10	High
Laffaye et al.	1	1	1	2	1	1	0	1	0	1	1	1	0	11	High
Floría et al.	1	1	1	2	1	0	0	1	0	1	1	0	0	9	Moderate
Bridgeman et al.	1	0	1	1	1	1	0	0	0	1	1	0	0	7	Moderate
González-Moro et al.	1	0	1	1	1	1	1	0	0	1	1	0	0	8	Moderate
Douglas et al.	1	1	1	2	1	1	0	1	0	1	1	1	0	11	High
Merino-Muñoz et al.	1	1	1	1	1	1	1	0	0	1	1	1	0	10	High
Harper et al.	1	1	1	1	1	0	0	1	0	1	1	0	0	8	Moderate
Krzyszkowski et al.	1	1	1	2	1	1	1	1	0	1	1	1	0	12	High
Harry et al.	1	1	1	2	1	1	1	0	0	1	0	1	0	10	High
Barker et al.	1	1	1	2	1	1	0	0	0	1	1	1	0	10	High
McHugh et al.	1	1	1	2	1	1	1	1	0	1	1	0	0	11	High
Oh and Lee	1	1	1	2	1	1	1	0	0	1	1	0	1	11	High

### Synthesis of the results

#### Eccentric variables

As eccentric variables during the CMJ, five forces (unloading-yielding, braking, unloading, amortization, and minimum forces) and three RFDs (braking, eccentric, and unloading RFDs) were reported. The other reported variables included time in four studies (unloading-yielding, eccentric, unloading, and braking times), power in four (unloading-yielding peak and average powers, and braking peak and average powers), velocity in two (maximum negative and downward velocities), impulse in two (unloading-yielding and braking impulses), and eccentric work in one. Two studies reported the eccentric forces during squatting (eccentric and isoinertial eccentric forces, respectively). One study reported the isokinetic eccentric force of the quadriceps and hamstrings at angular velocities of 60°/s and 180°/s, one study reported the eccentric torque of the knee extension, and another reported the eccentric mean force during drop jumps at 20 and 40 cm box heights.

#### Jump performance

The CMJ height, RSImod, and drop jump RSI and height have been reported. The jump heights were calculated using the impulse-momentum method in six studies, flight-time method in four studies, and no description was given in three studies. On the other hand, the RSI and RSImod were calculated using “jump height divided by contact time” and “jump height divided by jump time,” respectively. The countermovement strategy was uncontrolled or not described in the articles, with the exception of one study [11]. The arm swing strategy was either with arm swing or hands on hips, except for one study [29].

### Main results

The eccentric CMJ variables associated with the CMJ height included time, impulse, and peak and average powers for the unloading-yielding and unloading-braking phases, respectively, in one study each; braking force in two studies; braking RFD in four; and downward phase velocity in two. The eccentric non-CMJ variables reportedly associated with CMJ height included the eccentric forces during squatting and knee extension in two and one studies, respectively. The eccentric CMJ variables related to the RSImod were the unloading time and force, eccentric work and RFD, amortization force, and braking RFD. The eccentric force during squatting was reportedly associated with the drop jump RSI in one study.

Conversely, the eccentric variables during the CMJ reportedly not associated with jump height were the eccentric RFDs in two studies, unloading force and RFD, each in one study, and braking RFD, eccentric work, and amortization force, each in one study. In addition, no relationship was reported between the eccentric knee flexion force and CMJ height. The unloading RFDs in two studies and eccentric RFD in one were the eccentric variables reportedly not associated with the RSImod. The jump height and RSI in drop jump were not associated with the eccentric mean force during the drop jump.

## Discussion

This systematic review selected and reviewed studies that investigated the associations of eccentric force variables during jumping and eccentric lower-limb strength with vertical jump performance. To the best of our knowledge, this is the first review on this topic. The force and RFD during the unloading-to-braking phase of the CMJ were related to the CMJ height in some studies but not in others. On the other hand, the RFD during the unloading-to-braking phase of the CMJ was related to the RSImod, and the eccentric strength was related to CMJ height.

### Countermovement jump height

This review included studies that found and those that did not find an association between the force or RFD during the unloading-to-braking phase and CMJ height. A theoretical explanation for this positive association is the increased early concentric force. According to Newton’s second law, jump height is determined by the concentric phase impulse; a greater force applied immediately before the concentric phase (i.e., braking to amortization phase) leads to a greater early concentric force and higher jump height [10]. On the other hand, a theoretical explanation for the lack of a relationship between the CMJ height and force or RFD during the unloading-to-braking phase could be the increased center-of-gravity ascent velocity during the late concentric phase, which would hamper force exertion. Although countermovement use increases the early concentric force, it also increases the center-of-gravity ascent velocity. If this increase in velocity occurs during the late concentric phase, force exertion may be hampered because of the characteristic force-velocity relationship observed in the muscles [30]. However, this has not been directly demonstrated in research and can only be inferred. Another possible explanation is that the countermovement depth was not controlled in previous studies. In a study comparing the unimodal and bimodal force-time curves of the CMJ, the unimodal group had a significantly shallower countermovement depth and higher braking phase metrics (i.e., force, RFD, power etc.) despite no significant difference in the CMJ heights between the two groups [24]. This indicates that countermovement strategies (i.e., depth and velocity) [31] affect the eccentric strength variables; the association between these variables and the CMJ height may not be accurately investigated without specifying the countermovement strategies. The studies that investigated the association between the force or RFD during the unloading-to-braking phase of the CMJ and CMJ height in this review used uncontrolled countermovement strategies. Controlled countermovement strategies should be used to investigate this relationship in future research. Arm swing strategies also affect ground reaction force during CMJ [32]. Previous studies mentioned in our study were confounded by those using arm swing and those not using arm swing. However, in each of the studies that used or did not use arm swing, some studies found an association between eccentric force variables and CMJ height, while others did not. This may suggest that the use or lack of use of the arm swing strategy was not involved in the association between eccentric force variables and CMJ height. On the other hand, the sample sizes of the studies that found no relationship [21, 23] (Merino-Muñoz et al., 2020 and Barker et al., 2018, n = 26 and 21, respectively) were small compared to those of the studies that found relationships [19, 29] and differences [20, 24] (n = 48, 273, 34, and 36, respectively). A low sample size may cause a type II error (i.e., misidentifying a significant difference as not being significant) [17]. Therefore, caution is needed in interpreting these results, as they are likely to have insufficient sample size. In addition to the force and RFD, the other variables associated with CMJ height include the unloading-to-braking phase power, downward center-of-gravity velocity, and eccentric time. Positive braking phase impulse is determined by a negative unloading-yielding phase impulse. The greater force and velocity obtained during the unloading-to-yielding phase (faster center-of-gravity descent) increase the braking phase impulse and force requirements. Simultaneously, they lead to a shorter time from the unloading-to-braking phase. As mentioned above, the force during the early concentric phase increases, which may be related to the CMJ height.

The eccentric forces during squatting and knee extension significantly correlate with CMJ height [11, 28]. However, no significant correlation has been reported between the eccentric knee flexion force and jump height [11]. Knee torque and power during the CMJ contribute to the jump height [33], which is reasonable, given the importance of knee strength for jumping performance. This was also the case for squatting, which was related to the greater torque exerted by the knee during this movement [34]. On the other hand, knee flexion muscle strength is not important because the knee action in jumping is extension. In addition, knee flexion muscle strength essentially indicates the hamstring strength, which may also contribute to the hip extensors’ strength. However, hip extensors work in tandem with other muscles, such as the gluteus maximus and adductor magnus [35]. Owing to all these reasons, knee flexor strength might not be associated with CMJ height. Muscle strength and muscle-tendon interactions are related to the countermovement utilization ability, and tendons should be stretched with minimal muscle fascicular elongation during the amortization phase [36]. A high eccentric muscle strength indicates a greater ability of the muscle to resist stretching and relation to the muscle power output using the stretch-shortening cycle (SSC) [37]. Furthermore, Cormie et al. showed that increased muscle strength contributes to an increased countermovement utilization ability [10]. The findings of this review may suggest a relationship between the eccentric muscle strength and jump height. In the context of training instruction, to increase jump height it is considered effective to not only increase concentric muscle strength, but also to engage in training to target eccentric muscle strength.

### Reactive strength index-modified

RSImod, an indicator of jump performance, has been recently studied [38, 39]. RSImod is calculated by dividing the CMJ height by the CMJ’s time (the time taken from the start of the action to the takeoff) [38] and is a good indicator of the ability to jump within a limited time, particularly when jumping vertically and against other athletes, such as in basketball [8]. Our results demonstrated a significant relationship between the RSImod and braking RFD, amortization force, eccentric work, and unloading force in CMJs, but not unloading RFD. Additionally, one study found a significant relationship between the RSImod and eccentric RFD, and another study reported no significant differences between the two [26]. Increased jump heights and shorter jump times are required to improve the RSImod. Improving the concentric phase impulse increases the jump height and, simultaneously, center-of-gravity ascent velocity, thereby reducing the concentric time. On the other hand, a larger force during the unloading-to-braking phase of the CMJ contributes to a shorter jump time because it improves the early concentric phase impulse, as mentioned above, and shortens the time of the unloading-to-braking phases [10]. In this context, a significant relationship between the RSImod and unloading-to-braking phase variables, as observed in this study, seems reasonable. In contrast, there was no significant relationship or difference between the RSImod and unloading RFD [8, 21], which is calculated by dividing the change in force from the start of the movement to the point at which the ground reaction force reaches a minimum by the time taken and can be interpreted as an indicator of unloading. Therefore, the results of this study suggest that unloading may not contribute to the RSImod. However, these study results have insufficient statistical power (0.34 and 0.13, respectively); thus, caution may be needed when interpreting them. In addition, Harry et al. (2018) reported no difference in the eccentric RFD between the good and poor RSImod groups [26]. However, they studied between-group comparisons with sample sizes of 6 and 9, respectively, with insufficient statistical power (0.06); therefore, caution should be exercised while interpreting their results. In addition, a recent study found that the shallower the countermovement depth, the greater the RSImod, although the jump height tends to slightly decrease [31]. This indicates that the RSImod is significantly affected by a countermovement strategy. Therefore, future studies should investigate the relationship between the eccentric variables and RSImod using a standardized countermovement strategy.

### Reactive strength index

A correlation exists between the eccentric force of the squat movement and the drop jump RSI [22]. In addition, a difference in drop jump height was found between the high and low knee extension eccentric torque groups [27]. The RSI is obtained by dividing the jump height by the contact time and is considered an indicator of the SSC ability [6]. A greater eccentric force reduces the time between landing and the concentric phase, which contributes to a shorter contact time. As mentioned earlier, eccentric muscle strength can be interpreted as the ability of a muscle to resist stretching forces, which may contribute to favorable muscle-tendon interactions (i.e., isometric muscle contraction during the amortization phase) [35]. On the other hand, no relationship has been reported between the eccentric force of the drop jump and drop jump RSI [25]. Harper et al. (2022) also reported a negative correlation between eccentric mean force and contact time during the drop jump. Despite the shorter contact time, RSI remained the same, which may have had a negative influence on jump height. The eccentric force of the drop jump is greatly influenced by the momentum at landing. Therefore, too much momentum at landing would deviate from the optimal muscle-tendon complex behavior in SSC (i.e., the muscle isometric contraction), stretching the muscle and negatively affecting the force output during the concentric phase [25]. In the future, the relationship between the RSI and eccentric strength variables of the ankle plantar flexors should be investigated, as the drop jump is primarily a contribution of the ankle joint movement [40].

The limitations of this study were that a direct cause-effect relationship could not be demonstrated between the eccentric strength variables and vertical jump performance and that the eccentric strength exercise disciplines and variables used varied between the studies; therefore, an integrative meta-analysis was not possible.

## Conclusion

The eccentric variables during the CMJ are associated with the RSImod; however, their relationship with jump height remains unclear. Although improving the eccentric variables of the CMJ improves the early concentric phase force, it may not necessarily contribute to the CMJ height. Future research should implement controlled countermovement strategies and investigate their relationship with jump height, as the countermovement depth and velocity affect the eccentric variables. Improving the eccentric muscle strength may also contribute to the vertical jump height because of the associations between the eccentric forces during knee extension and squatting with vertical jump height.

## Supporting information

S1 Checklist (DOCX)
